# 

**Published:** 1897-01

**Authors:** 


### CONTENTS.

ORIGINAL ARTICLES:
Veterinarians as Sanitarians. By CLAUDE D. MORRIS . . . . . . . 1
Acetanilid (or Antifebrin). By F. S. ALLEN, M.D., D.V.S. . . . . . 10
Bacteriology in Its Relation to Veterinary Science. By M. P. RAVENAL, M.D. . 12
Lymphangitis. By A. B. CAMPBELL, V.S. . . . . . . . . . . 15
Communicable Animal Diseases. By W. HERBERT LOWE, D.V.S. . . . . . 19
ABSTRACTS FROM FOREIGN JOURNALS:
Tubercle-bacilli in the Feces—The Inoculability of the Tuberculosis of Mammiferæ on the Gallinaceæ—Intestinal Antisepsis by Purgation—Syncope in a Dog for Five Hours as the Result of a Chloral and Morphine Mixture—Treatment of Hygromain in the Front Knees of Cattle—Poisoning of Cattle Through a Licking-stone (Salt-poisoning)—The Cause of Addison's Disease . . . 22–26
EDITORIALS:
A National Misfortune—Army Legislation—Colorado Veterinarians Seek State Recognition—Future Meetings of the U. S. V. M. A.—The Value of Wise Sanitary Measures—Closer Affi iation Demanded—What We Want to Know . . 27–33
NECROLOGY—Jose Maria Peralta, M.D., D.V.S. . . . . . . . . . . 33
NEW PUBLICATIONS:
Diseases of the Dog and their Treatment—The Veterinary Blue-book of New York—History of American Veterinary Medicine—Study of the Diseases of Poultry—Guide to Meat-inspection . . . . . . . . . . . . 33–34
LEGISLATION:
A Proposed Bill to Regulate the Practice of Veterinary Surgery, Medicine, and Dentistry in the State of Colorado . . . . . . . . . . 35
CORRESPONDENCE:
Recent Changes in the Personnel of the United States Bureau of Animal Industry . 39
CONTROL WORK:
Pennsylvania—Maryland—Illinois—South Africa—Canada . . . . 41–42
SOCIETY PROCEEDINGS:
Illinois State Veterinary Medical Association—Montreal Veterinary Medical Association—Keystone Veterinary Medical Association—Oneida County Veterinary Medical Society—New Hampshire Veterinary Medical Association—Chicago Veterinary Society—New York State Veterinary Medical Society—Missouri Valley Veterinary Medical Association . . . . . . . . . . 43–59
COMMENCEMENT EXERCISES—PERSONAL—GLEANINGS—LIGHTS AND SHADOWS—DRIFT OF THOUGHT . . . . . . . . . . . . . . 59–66

